# Prevalence of Congenital Color Vision Deficiency in Southern Taiwan and Detection of Female Carriers by Visual Pigment Gene Analysis

**DOI:** 10.3390/ijms242015247

**Published:** 2023-10-17

**Authors:** Hsi-Kung Kuo, Shih-Ting Tsao, Pei-Chang Wu

**Affiliations:** 1 Department of Ophthalmology, Kaohsiung Chang-Gung Memorial Hospital, Kaohsiung 833, Taiwan; 2 School of Medicine, Chang-Gung University, Taoyuan 333, Taiwan

**Keywords:** color vision deficiencies, visual pigment genes, prevalence, proton carrier, deutan carrier, polymerase chain reaction

## Abstract

This study aimed to investigate the prevalence of color vision deficiencies (CVDs) and determine whether carriers could be detected by analyzing the visual pigment genes. Materials and Methods: The data of students who underwent routine CVD screening using the Ishihara color test in Kaohsiung, Southern Taiwan were analyzed. Furthermore, the DNA samples of 80 randomly selected females and four obligate carriers were analyzed. The most upstream genes, downstream genes, and the most downstream genes in the red/green pigment gene arrays were amplified separately using polymerase chain reaction (PCR), and exon 5 of each gene was analyzed. The prevalence of congenital red–green CVD in this study was 3.46% in males and 0.14% in females. The PCR analysis of the first gene, downstream gene, and last gene revealed normal patterns in 73 normal cases. Seven unusual patterns were detected in two proton carriers and five deutan carriers. Among the randomly selected females, 8.8% (7/80) were CVD carriers. The prevalence of CVD among male Taiwanese students in this study was 3.46%. Female carriers of congenital CVD can be identified by molecular analysis of the visual pigment genes. The proportion of CVD carriers among the randomly selected females was 8.8%, which was slightly higher than expected and further studies are warranted.

## 1. Introduction

Normal human color vision is trichromatic, which means that any color can be reproduced using a mixture of the three primary colors. The physiological substrate of color vision comprises three classes of cone photoreceptors, namely, the blue, green, and red cones, which are known as the short-, medium-, and long-wavelength sensitive cones, respectively. The different classes of cones respond to a wide range of wavelengths of light, resulting in overlapping sensitivity curves [1,2]. Congenital red–green color vision deficiency (CVD) is the most common inherited vision disorder. Its prevalence may be as high as 8% in White males and 1.0% in White females, with variations between individuals of different ethnic origins and countries [3,4,5]. Red–green CVD is an X-linked recessive trait. The genes OPN1LW and OPN1MW encode the long (L) and middle (M) wavelength sensitive cone opsins, respectively. The L, long-wavelength, is associated with red and M, middle wavelength, with green. The female has two X-chromosomes, but the male has only one X-chromosome. Therefore, females are more likely to be carriers of CVDs than males. The most upstream gene, downstream gene, and the most downstream gene in the red/green pigment gene tandem arrays of the two X-chromosomes were amplified, followed by exon 5 of each gene, using polymerase chain reaction (PCR) in each female subject. The most downstream gene might be the most upstream gene, downstream gene, third gene, or further, according to the gene deletion or rearrangement in the tandem array. Oda’s paper showed the demonstrating figures. Medium-wavelength) photopigment genes lie in a tandem “array” on the X-chromosome; the L gene is the first in the array, while the M is the second. Among individuals with normal color vision, there is variability in the number of OPN1LW and OPN1MW genes. Although many individuals have arrays of three or more genes, wherein a single L gene is followed by one or more M genes, only the first two genes are thought to be expressed at sufficient levels to influence color vision [6,7]. Exon 5 in each pigment gene determines the spectral sensitivities of the gene products. If exon 5 of the green gene (G) is found in the most upstream gene, the individual is a protan; if exon 5 of the red gene (R) is found in the downstream genes, the individual could be a deutan. Moreover, if exon 5 of the green gene (G) is absent, the individual will be a deutan [8,9].

There are no new data regarding CVD prevalence in Taiwan reported. In this study, we reported the survey results of the color vision of school students in Kaohsiung, Taiwan, and analyzed the photopigment genes of randomly selected females in the laboratory.

## 2. Results

### 2.1. Part I: Survey of Color Vision of Students

During the 3-year survey period, Grade 1 to Grade 7 school students in Kaohsiung comprised 98,202 male and 91,381 female students. CVD was observed in 3400 males (3.46%) and 132 females (0.14%). The overall CVD prevalence was 1.86% (3532/189,583) (Table 1).

### 2.2. Part II: Detection of Female Carriers

For the detection of female carriers, we randomly enrolled 80 females (Table 2). Most participants were nurses and medical researchers. Their ocular status was good, except for the refractive error. Regarding the Ishihara color test results, four participants missed one plate, while one participant missed two plates. These results were considered within the normal limits in this study.

**Figure 1:**
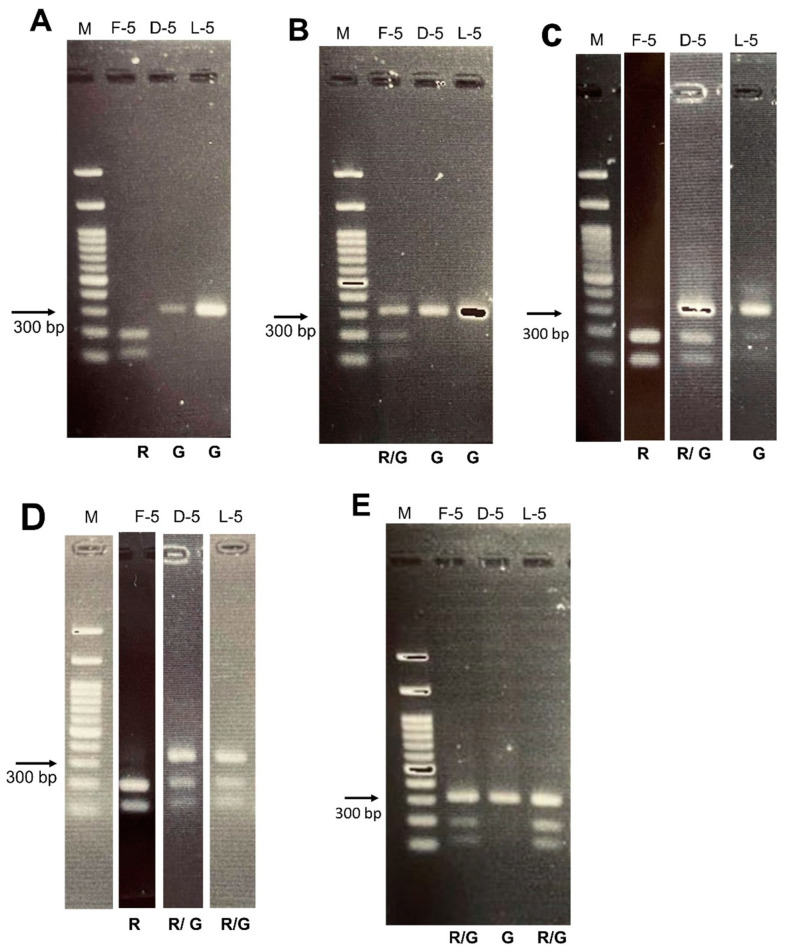
Two-step PCR performed for amplification of each exon 5 in first gene, downstream gene, and last gene of color vision on the 2 X-chromosome with two-stranded DNA in female subjects. Then, the DNA products were treated with RsaI enzyme and run with 2% agarose gels. The exon 5 of the red gene (R) can be cut to yield 2 fragments, but the exon 5 of the green gene (G) only yields 1 fragment. A to D, the gel pattern from the randomly selected female participant. (**A**), normal pattern (R-G-G). This means that R-G-G and R-G-G presented in the 2 X-chromosomes. (**B**), protan pattern (R/G-G-G). This means R-G-G and G-G-G in the 2 X-chromosomes. (**C**), deutan pattern (R-R/G-G). This means R-G-G and R-R-G in the 2 X-chromosomes. (**D**), deutan pattern (R-R/G-R/G). (**E**), Obligate carrier with protan pattern (R/G-G-R/G). M: marker. F-5: exon 5 in first gene. D-5: exon 5 in downstream gene. L-5: exon 5 in last gene. R: red gene, G: green gene.

PCR analysis of the first gene, downstream gene, and last gene revealed R-G-G in 73 normal cases (Figure 1A). There were seven unusual patterns detected, including two L gene defects, two M gene defects, three distal gene defects, two proton carriers, and five deutan carriers (Figure 1B–D). The gene arrays of these seven cases were (R/G-G-G) × 2, (R-R/G-R/G) × 1, (R-R/G-G) × 1, and (R-G-R/G) × 3. The proportion of CVD carriers was 8.8% (7/80). The five patients with minor errors in the Ishihara color test had normal PCR results.

Additionally, we enrolled four obligate carriers. The Ishihara color test results were normal in two obligate carriers, while the other two missed one plate (Table 2). The PCR showed an L gene defect in these four obligate carriers. Their gene arrays were (R/G-G-G) × 2, (R/G-G-X) × 1, and (R/G-G-R/G) × 1 (Figure 1E).

## 3. Discussion

The prevalence of congenital red–green CVD in a student population of Kaohsiung, Southern Taiwan was 3.46% in males and 0.14% in females. The prevalence in an aboriginal village in Taiwan was 3.62% among males. A similar percentage of CVD was observed in the aboriginal children and non-tribe students [10]. Previous studies reported a higher CVD prevalence in the White male population (6.6–8.3%) than in the Asian male (2.76–5.3%) and African male (4.2–4.8%) populations [1,3,4,5,11,12].The Multi-Ethnic Pediatric Eye Disease Study in Southern California, USA found a significant association between ethnicity and CVD. The prevalence of CVD was 1.4%, 3.1%, 2.6%, and 5.6% among Black, Asian, Hispanic, and non-Hispanic White male children, respectively; the prevalence among females was 0.0–0.5% for all ethnicities [5].

In this X-linked recessive hereditary disease, the incidence of CVD is higher in males than in females. Another recognized high prevalence occurs in genetically isolated populations. Similarly, Travellers in Ireland (21% male, 6.6% female), dental students in Abha, Saudi Arabia (20.19% male, 1.4% female), and the Meitei population in Manipur, India (14.93% male, 2.5% female) present higher CVD incidence than the average incidence [13,14,15]. The high prevalence could be due to the high frequency of consanguineous marriages in these populations [4,15]. Over the past 400 years, immigrants from different places of the world, especially Chinese, have inhabited Taiwan, which is an island in the West Pacific Ocean. Thus, a relatively low prevalence of congenital CVD on this open island is expected.

Those with CVDs are at a disadvantage when performing visual tasks and have traditionally been precluded from pursuing particular occupations. Early awareness of CVD is essential to avoid frustration in school learning and disappointment in career choices [1,16]. Family history is important for detecting and predicting CVD. Detection of female carriers is important for genetic consultation; however, only analyzing the visual pigment genes can achieve detection. The mothers of CVD patients are obligate carriers. Often in clinical scenarios, these mothers feel surprise and regret when they find out about the color vision problem in their children and the cause of this disease. Identifying CVD carriers could provide information on hereditary genetics and benefit genetic consultation. Female members of families with CVD as well as those without information about the color vision status of their family were included in this study. Analyzing the L/M visual pigment genes in females is difficult because of the dual gene arrays. Kainz et al. first described a method to survey the most upstream position of photopigment gene arrays for identifying protan carriers [17]. The methods for analyzing the proximal gene, downstream genes, and distal gene in the pigment gene array for female carrier detection were reported by Oda et al. [8,9]. In this study, we followed the previously described methods with minor modifications.

Owing to the proximity of the red and green cone photopigment genes and their high degree of similarity, the gene array is subject to unequal recombination (rearrangement). This may result in the deletion of whole genes or the generation of so-called red–green hybrid genes [6]. Such genotype–phenotype relationships established using genomic DNA samples have both false-positive and false-negative results. Several studies have reported red exon 5 in the downstream genes of males with normal color vision. The incidence was higher (3.1–8.0%) in White males with normal color vision than in their Japanese (0.9%) counterparts [18,19,20,21]. On the other hand, CVD individuals sometimes have a normal pigment gene arrangement (5.6%) [22]. Rarely, the photopigment genes or their promoter regions may contain point mutations [20,22,23,24]. These may be missed and show a false-negative result. Recent bulk transcriptomics studies could represent a strong substrate to enforce the role of described molecular mechanisms [25,26,27]. Although modern sequencing techniques have progressed quickly and multiple mutation disorders have been unlocked, knowledge about CVD inheritance has not improved much in recent years. Based on previous studies, most color vision defects were the result of gene rearrangements. A less frequent cause of inherited red–green color vision deficiencies had arisen through mutational mechanisms (4.3% male protans) [23]. The two-step PCR method was published 20 years ago. Genotype–phenotype relationships established by this method did have both false-positive and false-negative results but in very small proportions. Based on the study by Oda et al., the proportion of supposed deutan carriers (8.5%) was slightly higher than expected (7%) among the randomly selected females [8]. The estimated proportion of CVD carriers was 8.8%, and the prevalence of CVD was 3.46% in the males in our study. The supposed carrier proportion was also slightly higher than expected. Nevertheless, screening for female carriers of congenital CVD appears to be clinically practical.

The contribution of this study is providing the prevalence of CVD and the feasibility of genetic screening in the Taiwanese population. This is the first report of the prevalence of CVD in the Taiwanese population in the English literature. One of the limitations of our study is that we did not differentiate CVD into protanopia and deuteranopia. The Ishihara color test, which was used to identify CVD, is very sensitive in detecting CVD but less successful in differentiating between protans and deutans [4,28]. Moreover, the differentiation of the CVD types was not the purpose of this screening. Second, this survey of obligate carriers showed that all four individuals had the L gene anomaly. Usually, the M gene anomaly is more common than the L gene anomaly. Furthermore, our study sample size was small, and further studies with more cases are warranted.

In conclusion, the prevalence of CVD among Taiwanese students was 3.46% in males and 0.14% in females in this study. In randomly selected female subjects, the genetic survey was performed by using polymerase chain reaction to analyze the photopigment genes. The most upstream gene, downstream gene, and the most downstream gene in the red/green pigment gene tandem arrays followed by exon 5 of each gene were amplified. The proportion of CVD carriers among the randomly selected females was 8.8% from genetic survey. The molecular genetic analysis of photopigment genes using the two-step PCR method is clinically practical and feasible in the Taiwanese population. Further large-scale studies are warranted.

## 4. Materials and Methods

### 4.1. Part I: Survey of the Color Vision of Students

In Taiwan, the Ministry of Education requires students of Grades 1, 4, and 7 (age 7, 10, and 13 years old) to undergo annual health examinations, including physical examination, visual acuity test, color vision test, and laboratory tests. This study analyzed the data obtained from the examinations conducted between 2019 and 2021 in Kaohsiung, Southern Taiwan.

The school nurse administered the Ishihara color test to the school students to screen for CVD. The test included 17 plates, of which 1 demonstration plate and 16 test plates were used. The students were asked to identify the hidden number in each plate within 5 s. If there was an error in identifying the number missing two or more plates, the student was considered to have CVD and referred to an ophthalmologist.

### 4.2. Part II: Detection of Female Carriers

Women aged > 18 years were randomly selected for this study (*n* = 80). They did not have any major ocular disorders or a family history of CVD. The information of this research study was open to the public through the billboard of the hospital. We also enrolled obligate carriers (the mother of a male patient with CVD) as positive controls (*n* = 4). They were found through our CVD database. All consenting participants were recruited after approval from the Institutional Review Board of the Chang Gung Memorial Hospital (IRB number: 201802099B0).

#### 4.2.1. Color Vision Screening Tests

The participants underwent general ocular examinations, including slit-lamp biomicroscopy and indirect ophthalmoscopy, to exclude major eye diseases. Following this, the Ishihara color test was administered to detect congenital CVD. The failure criterion for this test was missing two or more numbers on the plates.

#### 4.2.2. DNA Extraction

We collected 3 mL of venous blood from each participant. Genomic DNA was extracted from the venous blood using Quick-DNA Miniprep Plus Kit (Zymo Research, Irvine, CA, USA).

#### 4.2.3. Long-Range and Secondary PCR (Two-Step)

The most upstream gene, downstream gene, and the most downstream gene in the red/green pigment gene tandem arrays of the 2 X-chromosome were amplified, followed by exon 5 of each gene, using polymerase chain reaction (PCR) in each female subject. The most downstream gene might be the most upstream gene, downstream gene, 3rd gene or further, according to the gene deletion or rearrangement in the tandem array. Oda’s paper showed the demonstrating figures and we followed the methods described by Oda et al. [8,9]. First, we conducted a long-range PCR for the first gene, downstream gene, and last gene. The primers of these three genes are described in Table 1 of their study [9]. The sizes of the amplified DNAs were as follows: first gene product, 15.8 kb; downstream gene product, 14.4 kb; and last gene-TEX28 product, 27.5 kb. A second PCR for exon 5 was performed using 1/3000 of these products as the template with primers I4–I5 in a 100 μL-reaction mixture and purified using DNA Clean & Concentrator-5 (Zymo Research, Irvine, CA, USA) to 15 μL of DNA. The 314-bp DNA products were treated with *Rsa*I enzyme (New England Biolab., Ipswich, MA, USA) and cultured in 2% agarose gels to isolate the products. Exon 5 of the red gene (R) could be cut to yield two fragments, but not exon 5 of the green gene (G). Females have 2 X-chromosomes with 2 sets of tandem array which displayed a mixture pattern in 2% agarose gel.

## Figures and Tables

**Table 1:** Enrolment number, ratio, gender ratio, and prevalence of color vision deficiency of school students in Kaohsiung, Taiwan.

2019	Age	Students No. of Total	% Of CVD in Male	% Of CVD in Female	% Of CVD in Total
Grade 1	7	22,681	3.07%	0.12%	1.63%
Grade 4	11	18,494	3.41%	0.09%	1.80%
Grade 7	13	21,055	3.88%	0.09%	2.07%
2020					
Grade 1	7	21,218	3.25%	0.21%	1.78%
Grade 4	11	19,374	3.05%	0.14%	1.65%
Grade 7	13	20,956	3.41%	0.15%	1.85%
2021					
Grade 1	7	22,511	3.91%	0.25%	2.16%
Grade 4	11	23,231	3.29%	0.13%	1.77%
Grade 7	13	20,063	3.88%	0.12%	2.05%
2019–2021					
Total students		189,583	3.46%	0.14%	1.86%

CVD: color vision deficiency.

**Table 2:** Demographics, Ishihara color test results, and gene findings in the randomly selected female subjects.

	Sex	Age	Ishihara Color Test	Gel
OC1	F	61	miss 1 plate	R/G-G-R/G
OC2	F	40	normal	R/G-G-X
OC3	F	40	normal	R/G-G-G
OC4	F	55	miss 1 plate	R/G-G-G
RC1	F	34	normal	R/G-G-G
RC2	F	37	normal	R-R/G-R/G
RC3	F	34	normal	R-G-R/G
RC4	F	30	normal	R-R/G-G
RC5	F	31	normal	R-G-R/G
RC6	F	51	normal	R-G-R/G
RC7	F	38	normal	R/G-G-G
N (*n* = 73)	F	37.48 (20~48)	4 miss 1 plate, 1 miss 2 plate	R-G-G

OC: obligate carrier; RC: random selected carrier; N: normal; F: female; R: red gene; G: green gene.

## Data Availability

Not applicable.
